# Cellular Proliferation of Equine Bone Marrow- and Adipose Tissue-Derived Mesenchymal Stem Cells Decline With Increasing Donor Age

**DOI:** 10.3389/fvets.2020.602403

**Published:** 2020-12-10

**Authors:** Jasmin Bagge, James N. MacLeod, Lise C. Berg

**Affiliations:** ^1^Department of Veterinary Clinical Sciences, University of Copenhagen, Copenhagen, Denmark; ^2^Maxwell H. Gluck Equine Research Center, Department of Veterinary Science, University of Kentucky, Lexington, KY, United States

**Keywords:** horse, aging, donor age, mesenchymal stem cells, proliferation, tumor suppressors

## Abstract

**Background:** Bone marrow (BM)- and adipose tissue (AT)-derived mesenchymal stem cells (MSCs) are used increasingly for autologous cell therapy in equine practice to treat musculoskeletal and other injuries. Current recommendations often call for 10–100 million MSCs per treatment, necessitating the expansion of primary cells in culture prior to therapeutic use. Of concern, human and rodent studies have shown a decline of both MSC recovery from sampled tissue and *in vitro* proliferative capacity with increasing donor age. This may be problematic for applications of autologous cell-based therapies in the important equine demographic of older patients.

**Objectives:** To investigate the effect of donor age on the cellular proliferation of equine BM- and AT-MSCs.

**Study Design:** *In vitro* study.

**Methods:** BM- and AT-MSCs and dermal fibroblasts (biological control) were harvested from horses in five different age groups (*n* = 4, *N* = 60); newborn (0 days), yearling (15–17 months), adult (5–8 years), middle-aged (12–18 years), and geriatric (≥22 years). Proliferation of the cells was tested using an EdU incorporation assay and steady state mRNA levels measured for targeted proliferation, aging, and senescence biomarkers.

**Results:** The cellular proliferation of equine BM- and AT-MSCs declined significantly in the geriatric cohort relative to the younger age groups. Proliferation levels in the two MSC types were equally affected by donor age. Analysis of steady state mRNA levels showed an up-regulation in tumor suppressors, apoptotic genes, and multiple growth factors in MSCs from old horses, and a down-regulation of some pro-cycling genes with a few differences between cell types.

**Main Limitations:** Potential age-dependent differences in cell function parameters relevant to cell-therapy application were not investigated.

**Conclusions:** The cellular proliferation of equine BM- and AT-MSCs declined at advanced donor ages. High levels of *in vitro* proliferation were observed in both MSC types from horses in the age groups below 18 years of age.

## Introduction

Mesenchymal stem cells (MSCs) have shown potential to facilitate the repair of certain musculoskeletal and other tissue injuries, and are being used increasingly in equine practice (1–3). Cellular proliferation has been shown to be positively correlated with regenerative potential (4). Bone marrow (BM)- and adipose tissue (AT)-derived MSCs are currently the choice of therapy, where BM-MSCs have shown higher potential to treat cartilage and bone injuries (5, 6). For successful therapies, a substantial number of cells are needed, which often requires extensive *ex vivo* cell expansion prior to implantation. Generally, cell-based therapy protocols call for 10–100 million MSCs per treatment and are typically used for clinical applications at passage 3–4 (7–10). Additionally, repeated MSC applications have shown beneficial effects *in vivo*, which further increases the need for cells (11).

Unfortunately, human and rodent studies have shown both a decline in recovery from sampled tissues and a drop in the *in vitro* proliferative capacity of BM- and AT-MSCs with increasing donor age (12–15). The frequency of BM-MSCs is lower compared to AT-MSCs at isolation (16, 17). MSCs from aged donors have also been determined to have increased expression of cell cycle arrest genes like p53 and p21 (12, 14, 18), and decreased expression of growth factors like vascular endothelial growth factor (VEGF) and insulin-like growth factor (IGF) (13).

Very little is known about these age-dependent relationships in horses. No equine BM-MSC donor age-dependent studies have been reported with multiple age groups to provide thresholds relevant for clinical practice, or have investigated if different MSC types are equally affected by donor age (16, 18–20). Expansion of cell numbers invariably causes a treatment delay, which may be an issue for some applications where early treatment has been shown to be beneficial (21). The inability to even achieve cell numbers recommended for therapy would likely have a far greater impact. Together, this may limit the clinical potential of MSCs from aged horses.

Presently, autologous treatment is preferred over allogenic treatment due to the risk of immunological reactions associated with allogenic treatments in equine models (22, 23). Clinical issues that may benefit from cell-based therapies occur across the full range of equine ages, including orthopedic problems in older sport and recreational horses (24). Together, this emphasizes the significance of understanding the effect donor age has on the proliferative capacity of equine MSCs.

The current study was, therefore, designed to test the hypothesis that increasing donor age is a major variable impacting equine BM- and AT-MSC proliferation with decreasing capacities. The aim of this study was to compare cellular proliferation and the expression of genes known to regulate cell proliferation in BM- and AT-MSCs from horses in five different age groups, and to test if the two MSC types were equally affected by donor age.

## Materials and Methods

### Experimental Samples

Three different cell types, BM-MSCs, AT-MSCs, and dermal fibroblasts (DF) (biological non-stem cell control), were harvested as detailed below immediately post-mortem from horses of mixed breeds across five age groups. Four donor horses were used for each cell type and age group. The age groups were newborn (0 days old), yearling (15–17-month old), adult (5–8-year old), middle-aged (12–18-year old), and geriatric (≥22-year old). DFs were chosen as a biological control due to their morphology and to have a non-stem cell type for comparison to potential MSC age-related changes. All horses were euthanized due to reasons unrelated to the current study. No systemic illness was apparent in any of the subjects, with the exception of some old horses as noted (Table 1). The study was conducted according to the ethical guidelines of animal research at the University of Copenhagen and the University of Kentucky. A written informed consent was obtained from all privately-owned horses prior to sample collection. Sample size was determined by two-sample *t*-test power analysis in R (version 3.6.0, The R Foundation for Statistical Computing, Vienna, Austria) using MSC proliferation pilot data (not shown) from newborn and geriatric horses. The power was set to 0.80 and the minimum relevant difference in proliferation rate was set to 10% between study groups. Experiments were performed at two different universities. An inter-laboratory control of BM-MSCs from the same yearling cell line was tested for proliferation rate and steady state mRNA levels at both sites after shipping RNA on dry-ice from one laboratory to the other to perform gene expression analyses on the same machines. No indication of significant differences between laboratories was found (data not shown). Plasticware, culture medium, reagents, commercially available kits, and protocols were kept constant throughout the entire study for all samples.

**Table 1:** Overview of biological replicates used as study population.

**Age group**	**Age**	**Breed**	**Gender**	**BM-MSCs**	**AT-MSCs**	**DFs**	**Reason for euthanasia**
Newborn	0 d.	Pony	M	Yes	Yes	Yes	Research
	0 d.	Pony	M	Yes	Yes	Yes	Research
	0 d.	Pony	M	Yes	Yes	Yes	Research
	0 d.	Pony	F	Yes	Yes	Yes	Research
Yearling	15 mo.	Mixed, light breed	M	Yes	No	Yes	Research
	15 mo.	Mixed, light breed	M	No	Yes	Yes	Research
	15.5 mo.	Mixed, light breed	M	Yes	No	No	Research
	16 mo.	Mixed, light breed	F	Yes	Yes	Yes	Research
	16 mo.	Mixed, light breed	F	Yes	Yes	Yes	Research
	16 mo.	Mixed, light breed	M	No	Yes	No	Research
Adult	5 yo.	Standardbred	F	Yes	Yes	No	Research
	5 yo.	Standardbred	F	No	No	Yes	Research
	6 yo.	Standardbred	F	No	No	Yes	Research
	6 yo.	Standardbred	F	No	No	Yes	Research
	7 yo.	Standardbred	F	Yes	Yes	No	Research
	7 yo.	Standardbred	M	Yes	Yes	Yes	Research
	8 yo.	Warmblood	M	Yes	Yes	No	Unknown
Middle-aged	12 yo.	Standardbred	F	No	No	Yes	Research
	12 yo.	Standardbred	F	No	No	Yes	Research
	13 yo.	Standardbred	F	Yes	Yes	No	Research
	14 yo.	Pony	M	No	Yes	No	Unknown
	15 yo.	Standardbred	F	No	Yes	No	Research
	15 yo.	Standardbred	F	Yes	No	No	Research
	16 yo.	Pony	M	No	Yes	No	Unknown
	16 yo.	Thoroughbred	F	Yes	No	Yes	Research
	18 yo.	Thoroughbred	F	Yes	No	Yes	Weight loss
Geriatric	22 yo.	Thoroughbred	F	Yes	Yes	Yes	Research
	25 yo.	Coldblood	M	Yes	Yes	No	Unknown
	25 yo.	Thoroughbred	F	Yes	Yes	Yes	Lymphoma
	31 yo.	Thoroughbred	F	Yes	Yes	Yes	Colic
	32 yo.	Thoroughbred	F	No	No	Yes	Research

* *Research horses were euthanized for reasons unrelated to the current study and all were in apparent good health at the time of sacrifice. d., days old; mo., months old; yo., years old; M, male; F, female; BM, bone marrow; AT, adipose tissue; MSCs, mesenchymal stem cells; DF, dermal fibroblasts*.

### Bone Marrow Derived MSC Collection and Isolation

BM was collected immediately post-mortem from the sternum of 12 female and 8 male horses (Table 1).

For newborns, BM was collected as described by Vidal et al. (16) with a few modifications. Briefly, BM samples were obtained from the 4th−6th sternebrae by sterile curettage after splitting the sternum along the midline. The marrow trabecular bone was transported on ice to the laboratory in sterile Dulbecco's phosphate buffered saline (dPBS, Thermo Fisher Scientific, Waltham, MA, USA) with 2% (v/v) amphotericin B (Thermo Fisher Scientific, Waltham, MA, USA) and 2% (v/v) penicillin/streptomycin (P/S, Thermo Fisher Scientific, Waltham, MA, USA) (isolation solution) and processed within 2 h of collection. Marrow trabecular bone was rinsed twice in 37°C isolation solution and crushed before being grown as explant culture in two T75 flasks (Cellstar BioGreiner tissue culture treated flasks, Sigma-Aldrich, St. Louis, MI, USA) with 12 mL/flask Dulbecco's modified Eagle's medium (Gibco DMEM, 1 g/L glucose, with phenol red, GlutaMAX, and pyruvate, Thermo Fisher Scientific, Waltham, MA, USA), 10% (v/v) fetal bovine serum (FBS, Thermo Fisher Scientific, Waltham, MA, USA), 1% (v/v) P/S, and 1% (v/v) amphotericin B (isolation medium). After 48 h, BM crusts and non-adherent cells were aspirated along with the isolation medium, and the medium was changed to expansion medium containing DMEM, 10% FBS, and 1% P/S.

For the four older age groups, 20 mL of BM aspirate was collected from the 4th−6th sternebrae with a 11G Jamshidi® BM needle (Henry Schein Vet, Dublin, OH, USA) and BM-MSCs were isolated with Ficoll-Paque® PREMIUM (GE Healthcare, Chicago, IL, USA) as previously described (16, 25).

The method used for sample collection of BM-MSCs varied between newborns and the other age groups because the smaller size and cartilaginous sternum of newborn foals proved challenging for the accurate positioning of Jamshidi® needles to obtain high quality bone marrow aspirates.

### Adipose Tissue Derived MSC Collection and Isolation

AT was collected and isolated from the gluteal region above the biceps femoris muscle next to the tail base of 10 female and 10 male horses (Table 1) as previously described (25).

Briefly, the gluteal area next to the tail base was surgically clipped and prepared, and 10 grams of AT was collected through a ~8 × 8 cm surgical window. AT was transferred to a 50 mL polypropylene tube (Falcon) with ice cold isolation solution and transported on ice to the laboratory, where the tissue was further processed within 2 h of collection. AT was washed two times in isolation solution and dissected into smaller pieces where visible blood vessels were removed. AT was digested in sterile filtered (0.2 μm) enzyme medium consisting of DMEM (1 g/L glucose), 1% (v/v) P/S, 50 μg/mL gentamycin (Sigma-Aldrich, St. Louis, MI, USA), and 1 mg/mL collagenase type I (Thermo Fisher Scientific, Waltham, MA, USA) for 3 h at 37°C and 30 rpm. Released cells were filtered through a 70 μm cell strainer, washed twice in sterile dPBS, and centrifuged at 500 g for 5 min between the washes. The pellet was then re-suspended in 24 mL isolation medium supplemented with 50 μg/mL gentamycin and distributed into two T75 flasks. Medium change to expansion medium occurred 48 h after isolation.

### Dermal Fibroblast Collection and Isolation

Unfortunately, DFs had not been collected originally from all donor horses for which archived primary BM and AT cell lines were available. To include this biological control cell type in the experimental design of the current study, additional donors were recruited in the appropriate age group as needed. DFs were harvested and isolated from the gluteal region above the biceps femoris muscle next to the tail base of 14 female and 6 male horses (Table 1) as previously described (6).

In short, approximately 6 grams of dermal tissue was collected from either the same surgical window generated to collect AT-MSCs or one comparably positioned. Dermis was transferred to a 50 mL polypropylene tube (Falcon) with ice cold isolation solution and transported on ice to the laboratory where the tissue was further processed within 2 h of collection. The dermal tissue was washed two times in isolation solution, dissected into smaller pieces, and digested in sterile filtered (0.2 μm) enzyme medium consisting of dPBS, 1% (v/v) P/S, 1% (w/v) bovine serum albumin (BSA, Sigma-Aldrich, St. Louis, MI, USA), 50 μg/mL gentamycin, and 1 mg/mL collagenase type I for 2 h at 37°C and 30 rpm. Released cells were filtered through a 70 μm cell strainer, washed twice in sterile dPBS, and centrifuged at 1,000 g for 4 min between the washes. The pellet was then re-suspended in 24 mL isolation medium supplemented with 50 μg/mL gentamycin and distributed into two T75 flasks and grown at 37°C in air with 5% CO_2_. A culture medium change to expansion medium was performed 48 h after isolation.

### Cell Expansion and Storage

The cells were cultured in expansion medium at 37°C in a humidified atmosphere containing 5% CO_2._ Expansion medium was changed every 2–3 days. At approximately 80% confluence, the cells were passaged with Trypsin/EDTA (Thermo Fisher Scientific, Waltham, MA, USA). Cell counting was performed manually using trypan blue (Thermo Fisher Scientific, Waltham, MA, USA) and a hemocytometer. The cells were grown with a seeding density of 500,000 cells per T75 flask. At passage 2, the cells were cryopreserved at a concentration of 2–3 million cells/mL freezing medium (Recovery-Cell Culture Freezing Medium®, Thermo Fisher Scientific, Waltham, MA, USA) in cryogenic vials (Nalgene, Thermo Fisher Scientific, Waltham, MA, USA).

For subsequent experimental applications, the cells were thawed in 37°C expansion medium and washed three times in dPBS before being plated and grown in expansion medium. As before, the cells were incubated at 37°C and 5% CO_2_ with a medium change every 2–3 days. Proliferation and gene expression analyses were conducted with passage 4 cells.

### Assessment of Cellular Proliferation

Cellular proliferation was quantified by determining levels of incorporated 5-ethynyl-2′-deoxyuridine (EdU) after pulse labeling using a Click-iT Plus Alexa Fluor 594 EdU Imaging Kit® (Thermo Fisher Scientific, Waltham, MA, USA) as described previously (19).

In short, passage 4 cells were seeded at a density of 25,000 cells per well in a 24-well plate (Thermo Fisher Scientific, Waltham, MA, USA) and cultured in expansion medium for 48 h. The cells were then pulsed for 24 h with 8 μM EdU (Jena Bioscience, Jena, Germany). A total of eight technical replicate wells were pulsed with EdU, and one control well was kept under normal expansion medium without pulsing. Next, the cells were fixed in 4% methanol-free paraformaldehyde (Thermo Fisher Scientific, Waltham, MA, USA) and washed with 3% (w/v) BSA (Sigma-Aldrich, St. Louis, MI, USA) before being permeabilized with 0.1% Triton-X (Sigma-Aldrich, St. Louis, MI, USA). For EdU label detection, the cells were incubated for 30 min with the kit reagent cocktail containing Alexa 594. The staining cocktail was removed and the cells were washed with 3% BSA and dPBS before being counterstained with 4′,6-diamidino-2-phenylindole dihydrochloride (DAPI, Thermo Fisher Scientific, Waltham, MA, USA) for 15 min at a concentration of 1 μg/mL. The fluorophore staining cocktail was prepared fresh for each assay and the cells were incubated while protected from light. Images of the cells were taken in the dark using a fluorescence microscope with DAPI and Alexa 594 filters. A total of three random images were taken per well for each fluorophore. The total number of cell nuclei and the number of proliferating cells were counted using automated imaging software (Image-J version 1.48, NIH, Bethesda, MD, USA). The cellular proliferation rate was calculated as the number of EdU labeled nuclei as a percentage of total cell nuclei in each image. The proliferation percentage was calculated for all three images per well and then averaged.

### Differential Gene Expression

#### RNA Isolation and Reverse Transcription

Passage 4 cells were expanded in T75 flasks with expansion medium. At approximately 80% confluence, the cells were extracted with QIAzol® (Qiagen, Germantown, MD, USA) and snap frozen in liquid nitrogen before being stored at −80°C prior to RNA isolation. The cells were homogenized with a PowerGen 125 homogenizer (Thermo Fisher Scientific, Waltham, MA, USA). Total RNA was isolated using a Qiagen RNeasy Mini Kit® (Qiagen, Germantown, MD, USA) with modifications as previously described (26). RNA quantity was estimated with a NanoDrop spectrophotometer (Thermo Fisher Scientific, Waltham, MA, USA) prior to ethanol precipitation. Purified RNA was quantified using a Qubit BR Assay (Thermo Fisher Scientific, Waltham, MA, USA) and NanoDrop spectrophotometer. The quality of the purified RNA was assessed with a Bioanalyzer 2100 (Eukaryotic Total RNA Nano & Pico Series II, Agilent Technologies, Santa Clara, CA, USA). All purified RNA samples met the following quality thresholds; 260/280 ratios of 1.9–2.1, 260/230 ratios of 1.8–2.28, and an Agilent RNA integrity number (RIN) of ≥8.0, with the exception of one sample (RIN = 7.5) that behaved as expected in down-stream analyses and thus was not excluded from the study. Removal of potential genomic DNA contamination and reverse transcription of total RNA to cDNA was achieved using a commercially available kit as per manufacturer's protocol (Maxima First Strand cDNA Synthesis Kit® for RT-qPCR with dsDNase, Thermo Fisher Scientific, Waltham, MA, USA). All cDNA samples were diluted with nuclease-free water to 13.9 ng/uL and stored at −80°C.

#### Real-Time Quantitative PCR

Forty seven biomarkers were selected for gene expression analyses based on functional annotation. Selected gene loci were chosen due to important biological relevance for cell proliferation, cellular senescence, or evidence of age-dependent variation related to proliferation in the literature. Commercially available, validated equine-specific TaqMan® primer-probe sets (Thermo Fisher Scientific, Waltham, MA, USA) (Table 2) for all biomarkers were used to quantitate steady state mRNA levels. The functionality of all primer-probe sets was tested against a positive control equine sample containing mixed cDNA from equal amounts of a 43-sample pool of various tissues (27), day 35 whole fetus, and neonatal epiphyseal cartilage. Negative controls of RNase-free water and minus-template were incorporated, and each sample was run in duplicate. Real-time quantitative PCR (RT-qPCR) reactions were conducted in a 384-well plate with 62.55 ng cDNA per reaction using a robotic ViiA™ RT-qPCR System (Thermo Fisher Scientific, Waltham, MA, USA). LinRegPCR was used to measure reaction amplification efficiencies and cycle threshold (Ct) values were calculated (28). All targets yielded amplification efficiencies close to 2 except for the negative controls that showed no amplification as expected. Three commercially available equine-specific endogenous control TaqMan® primer-probe sets; β-2-microglobulin (B2M), β-glucoronidase (GUSB), and ribosomal protein lateral stalk subunit P0 (RPLP0), were tested against all samples. Using NormFinder software (Aarhus University Hospital, Aarhus, Denmark) (29), GUSB was determined as having the most uniform performance across all cell types and age groups.

**Table 2:** Overview of TaqMan primer-probe sets used in RT-qPCR reactions.

**Gene ID**	**Gene name(s)**	Gene function^*^	**ThermoFisher Assay ID**
ABI3BP	ABI family member 3 binding protein	Decrease proliferation	Ec06625599_m1
AQP1	Aquaporin	Increase proliferation	Ec06625425_m1
AREG	Amphiregulin	Mitogenic effect	Ec06992855_m1
BARD1	BRCA1-associated RING domain 1	Tumor suppressor	Ec07061151_m1
BAX	BCL2 associated X apoptosis regulator	Pro-apoptotic	Ec07016716_s1
BCL2	B-cell lymphoma 2	Anti-apoptotic	Ec07005800_g1
BMP3	Bone morphogenic factor 3	Increase proliferation	Ec07037656_m1
BRCA1	Breast cancer type 1	Tumor suppressor	Ec07017862_s1
CASP3	Caspase 3	Apoptotic gene	Ec03470391_m1
CASP8	Caspase 8	Apoptotic gene	Ec06959413_m1
CAVIN1	Caveolae associated protein 1	Induce senescence	Ec07036873_m1
CCND1	Cyclin D1	Pro-cycling gene	Ec07036996_m1
CDKN1A	p21, Cyclin-dependent kinase inhibitor 1A	Tumor suppressor	Ec06955195_m1
CLU	Clusterin	Anti-apoptotic	Ec03468575_m1
COMP	Cartilage oligomeric matrix protein	Increase proliferation	Ec03468062_m1
CSF2	Colony stimulating factor 2	Increase proliferation	Ec03468208_m1
CTGF	Connective tissue growth factor	Increase proliferation	Ec06625777_gH
CTNNB1	Beta-catenin	Increase proliferation	Ec00991819_m1
EPGN	Epithelial mitogen	Mitogen	Ec06992859_m1
FGF1	Fibroblast growth factor 1	Mitogen	Ec01092738_m1
FGF5	Fibroblast growth factor 5	Mitogen	Ec04656774_m1
FGF18	Fibroblast growth factor 18	Mitogen	Ec03248217_g1
GDF6	Growth differentiation factor 6	Tumor suppressor	Ec07097112_m1
GLB1	Beta-galactosidase	Senescence marker	Ec06954363_m1
GLI3	GLI family zinc finger 3	Regulate proliferation	Ec06625512_m1
HDGF	Hepatoma-derived growth factor	Mitogen	Ec07037751_m1
HGF	Hepatocyte growth factor	Mitogen	Ec07000054_m1
IGF1	Insulin-like growth factor 1	Mitogen	Ec03468689_m1
IGFBP5	Insulin-like growth factor binding protein 5	Mitogen binding	Ec03470296_m1
LOC100146270	p16, Cyclin-dependent kinase 4 inhibitor B	Senescence marker	Ec07037471_mH
MET	MET proto-oncogene, receptor tyrosine kinase	Regulate proliferation	Ec02622441_m1
MYC	c-myc	Pro-cycling	Ec07007511_m1
PCNA	Proliferating cell nuclear antigen	Increase DNA replication	Ec06974312_m1
PDGFD	Platelet-derived growth factor subunit D	Mitogen	Ec06997714_m1
PHB	Prohibitin	Inhibits DNA synthesis	Ec07055990_m1
PTCH2	Patched 2	Tumor suppressor	Ec06625424_g1
S100A1	S100 calcium binding protein A1	Inhibits proliferation	Ec03470173_g1
SNAI2	Snail family transcriptional repressor 2	Anti-apoptotic	Ec06625397_m1
SOST	Scherostin	Pro-apoptotic	Ec07036868_m1
TERT	Telomerase reverse transcriptase	Subunit of telomerase	Ec06972692_m1
TGFA	Transforming growth factor alpha	Regulate proliferation	Ec06949183_m1
TGFB1	Transforming growth factor beta 1	Regulate proliferation	Ec06625477_m1
TGFB2	Transforming growth factor beta 2	Regulate proliferation	Ec07074189_g1
TGFB3	Transforming growth factor beta 3	Regulate proliferation	Ec00682163_m1
TIMP2	Metallopeptidase inhibitor 2	Decrease proliferation	Ec03470558_m1
TP53	Tumor protein 53	Tumor suppressor	Ec03470648_m1
VEGFA	Vascular endothelial growth factor A	Mitogen	Ec03467879_m1
**B2M**	Beta-2-microblobulin	Endogenous control	Ec03468699_m1
**GUSB**	Beta-glucoronidase	Endogenous control	Ec03470630_m1
**RPLP0**	Ribosomal protein lateral stalk subunit P0	Endogenous control	Ec04947733_g1

* *NIH, Genetics Home Reference (https://ghr.nlm.nih.gov/gene/)*.

Steady state mRNA levels from the selected gene loci were determined by RT-qPCR using the BIOMARK HD System (Fluidigm Corporations, South San Francisco, CA, USA) as previously described (30) at a cDNA concentration of 13.9 ng/μL. Negative controls and seven dilutions of the positive control sample described above were incorporated, each dilution being 3-fold and ranging from 0.17 to 125 ng/μL. The Fluidigm protocol was carried out using the 96.96 Dynamic Array (Fluidigm Corporations, South San Francisco, CA, USA) according to manufacturer's instructions. Data were analyzed with Fluidigm Real-Time PCR Analysis Software in the BIOMARK instrument (Fluidigm Corporations, South San Francisco, CA, USA), where Ct values were calculated. Delta Ct values were determined for each sample by subtracting the corresponding Ct value of the endogenous control (GUSB). The positive control was used as a calibrator to calculate ΔΔCt values. Relative expression (RQ) of the gene targets were calculated using the 2^−ΔΔCt^ method (31). RQ levels were used for graphical bar/boxplot presentations made in GraphPad Prism (version 8.0.1, GraphPad Prism, San Diego, CA, USA), and Ln(RQ) values were used for heatmap and statistical analyses (Supplementary Table 1).

For visualization of relative transcript levels between experimental groups, a heatmap was generated from the averaged Ln(RQ) levels, grouping the samples according to cell type and donor age, and genes according to biological function. The heatmap was prepared in R (version 3.6.0, The R Foundation for Statistical Computing, Vienna, Austria) using the *heatmap ggplot* function.

### Statistical Analysis

The cellular proliferation data, comparing age groups within cell types and across cell types, were analyzed with a generalized linear mixed model using The GLIMMIX Procedure in SAS (version 9.4, SAS Institute Inc., Cary, NC, USA) with Tukey-Kramer's *post hoc* modifications for multiple comparisons. Gene expression data were analyzed in two steps. Initially, one-way analysis of variance (ANOVA) using SAS (version 9.4, SAS Institute Inc., Cary, NC, USA) was applied individually to all 47 gene targets within each cell type to look for donor age effects. Next, transcripts demonstrating significant differences by ANOVA and more than 5-fold difference between study groups on the heatmap were analyzed using The GLIMMIX Procedure with Tukey-Kramer's *post hoc* modifications for multiple comparisons to compare across tissue types. Normality of data was confirmed by QQ-plots and Shapiro-Wilk test. Statistical analyses of Fluidigm RT-qPCR results were performed on individual extracted Ln(RQ) values. Genes with missing data points (due to lack of detectable expression) were removed from the statistical analysis for the given cell type. To control for non-paired samples and potential inter-laboratory variables, horse number and laboratory were added to the statistical models as additional factors. Data were considered statistically significant at *p* < 0.05.

## Results

### EdU Proliferation Assay

**Figure 1:**
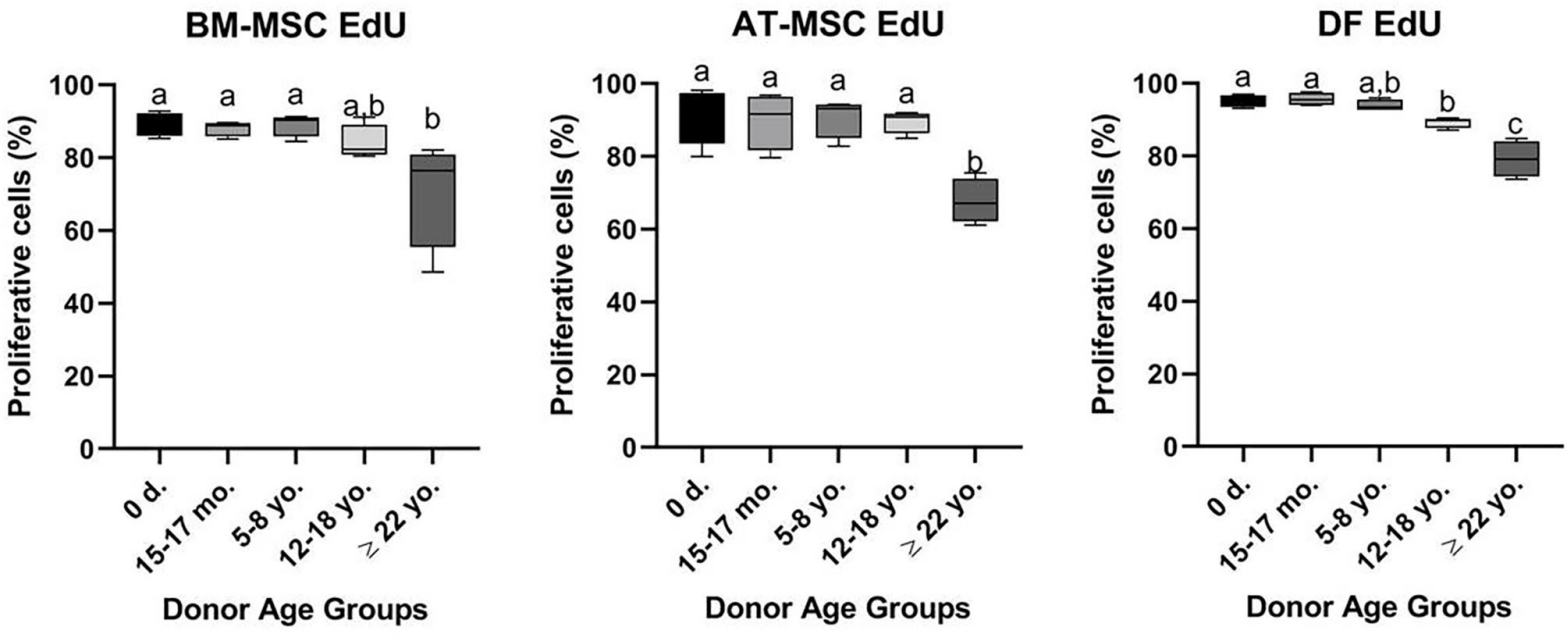
Age-dependent changes in cellular proliferation within each cell type. Box plots showing the cellular proliferation in percentage of bone marrow (BM)- and adipose tissue (AT)-derived mesenchymal stem cells (MSC) and dermal fibroblasts (DF) from horses in five different age groups (*n* = 4, *N* = 60) after 24 h of labeling with 8 μM EdU. Age groups within the same cell type not labeled with the same letter are significantly different from each other (*p* < 0.05). d., days old; mo., months old; yo., years old.

**Figure 2:**
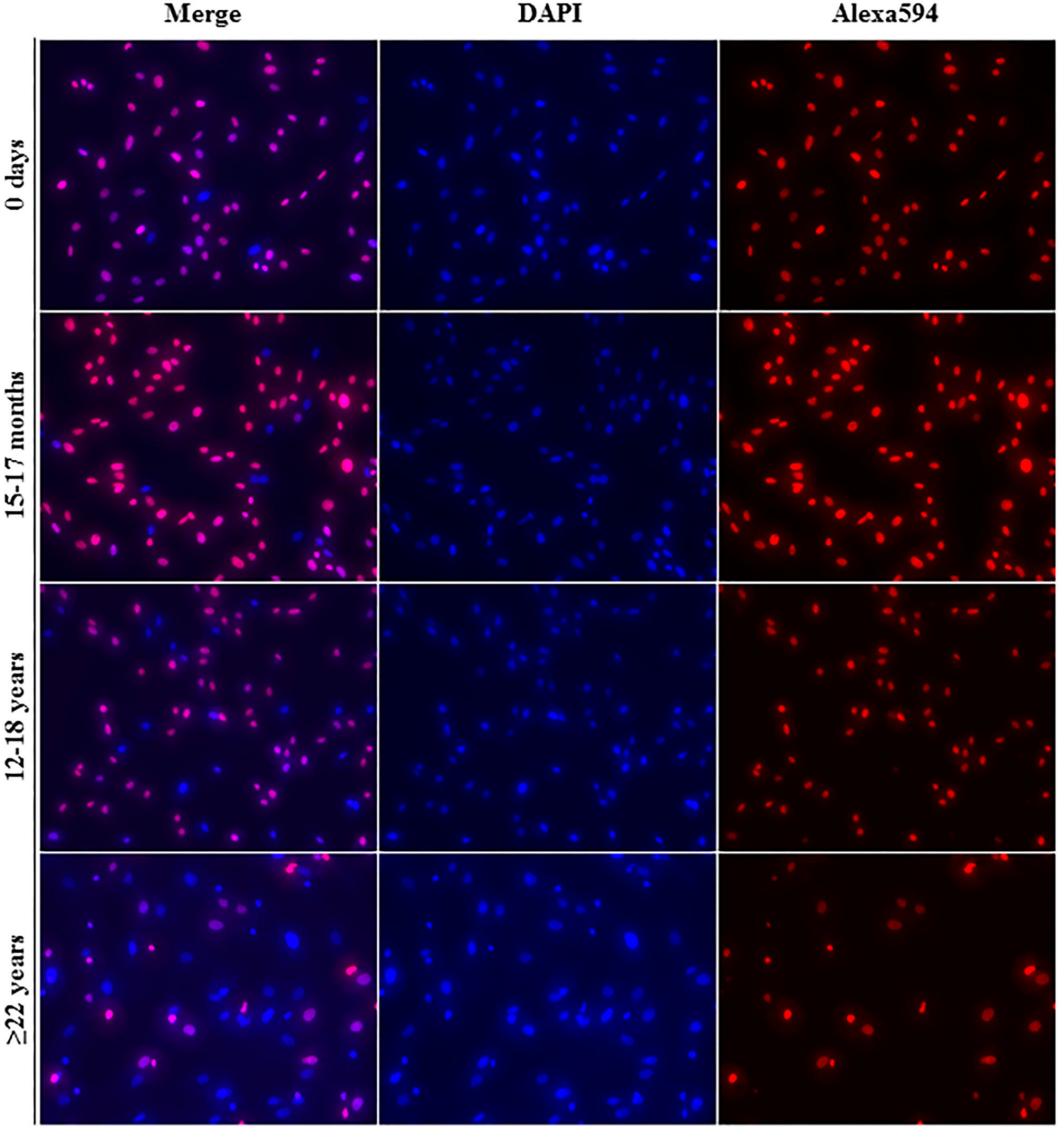
Fluorescence labeling. Representative images (20× magnification) of EdU-labeled proliferating cell nuclei (Alexa594—red/pink) and all cell nuclei (DAPI—blue) from equine bone marrow derived mesenchymal stem cells in different age groups.

All cell lines were able to be expanded to ~80% confluence at passage 4. When assessing the effect of donor age within one cell type, there was a significant decrease in cellular proliferation with increasing donor age for BM-MSCs (*p* = 0.02), AT-MSCs (*p* = 0.0003), and DFs (*p* < 0.0001) (Figure 1). Interestingly, there was no significant difference in pairwise comparisons between age groups other than geriatric horses for BM- and AT-MSCs (Figure 1). Representative images of proliferating BM-MSCs from horses in different age groups are shown in Figure 2.

**Figure 3:**
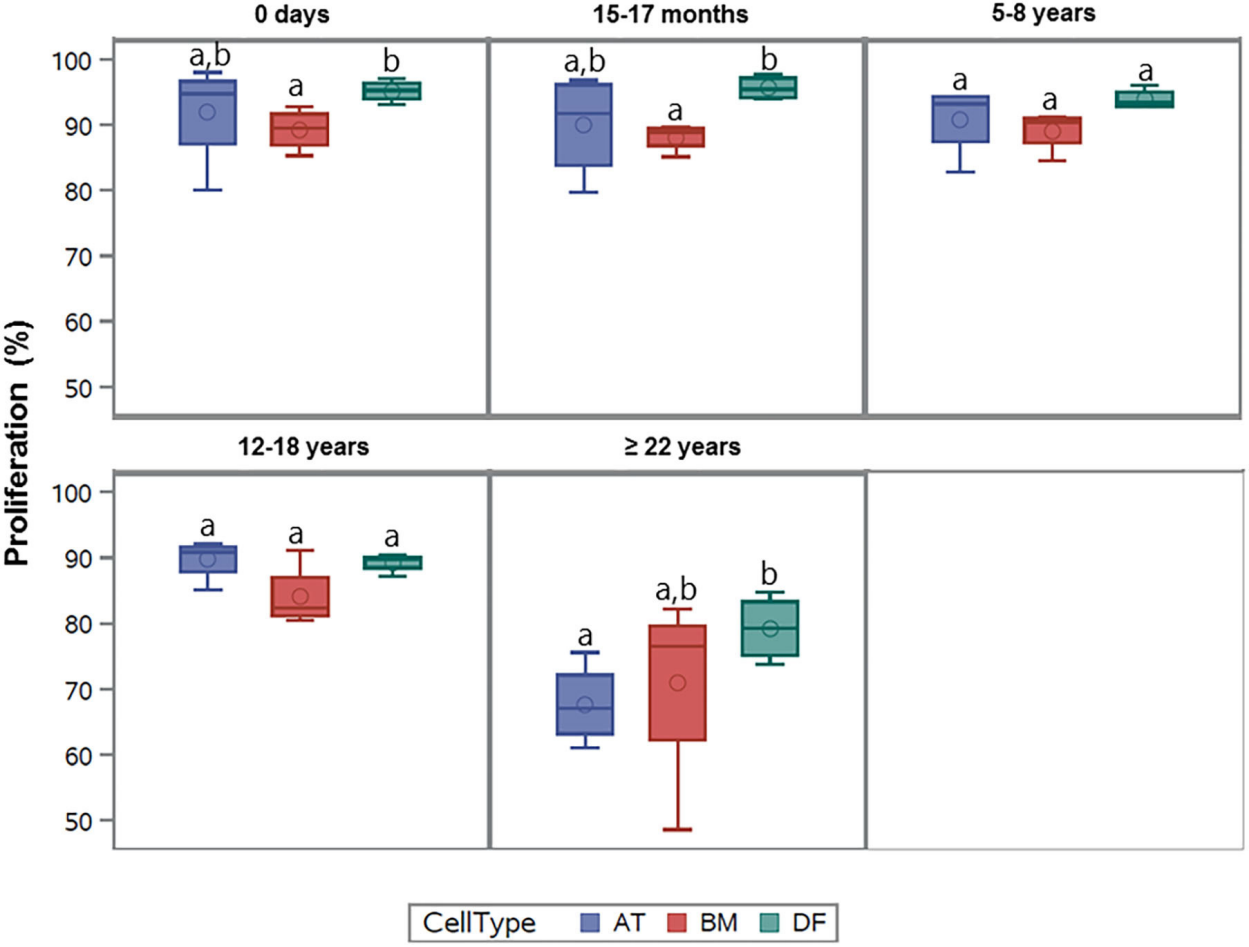
Age-dependent changes in cellular proliferation between cell types. Box plots showing the cellular proliferation (%) of equine adipose tissue (AT)- and bone marrow (BM)-derived mesenchymal stem cells and dermal fibroblasts (DF) from horses in five different age groups (*n* = 4 horses per age group per cell type) after 24 h of labeling with 8 μM EdU. Cell types within the same age group not labeled with the same letter are significantly different from each other (*p* < 0.05).

All cell types were equally affected by donor age (*p* = 0.3) and no significant differences were observed in cellular proliferation between BM- and AT-MSCs in any age group (*p* > 0.4) (Figure 3). When comparing cell types independent of age groups, DFs had a higher overall level of cellular proliferation relative to BM-MSCs (*p* = 0.006) and AT-MSCs (*p* = 0.02). No Alexa 594 background staining was detected in any of the negative control wells grown without EdU pulsing. No study group reached a cellular proliferation of 100% (Figure 1). Alexa 594 staining was detected in all wells pulsed with EdU, together with DAPI staining.

### Gene Expression

**Figure 4:**
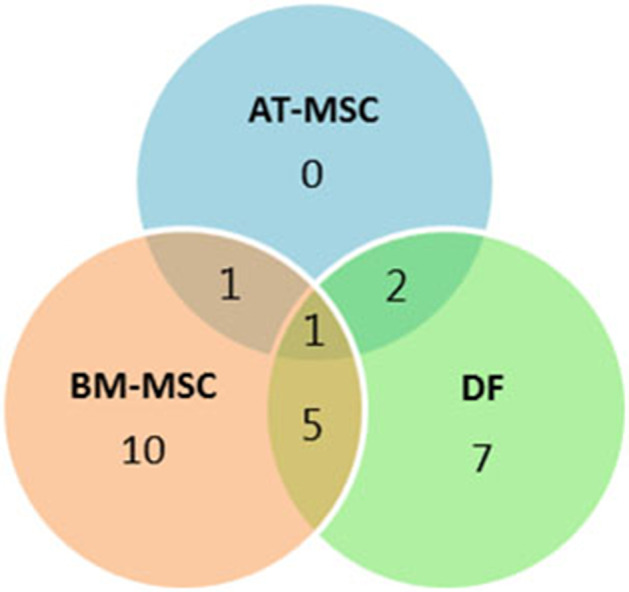
Concordance by cell type of differentially expressed genes. Venn-diagram showing the distribution of differentially expressed genes as a function of donor age between adipose tissue (AT)- and bone marrow (BM)-derived mesenchymal stem cells (MSC) and dermal fibroblasts (DF) from horses in five different age groups. The Venn-diagram represents data reported in Table 3.

Out of the 47 targeted gene loci related to proliferation and aging, steady state mRNA levels were affected by donor age in four biomarkers (9%) for AT-MSCs, 17 biomarkers (36%) for BM-MSCs, and 15 biomarkers (32%) for DFs. Differentially expressed genes as a function of donor age are shown in Table 3 and in a Venn-diagram in Figure 4 where intersections are visualized.

**Table 3:** Differentially expressed genes related to cellular proliferation as a function of donor age within adipose tissue- and bone marrow derived mesenchymal stem cells and dermal fibroblasts from horses in five different age groups (*n* = 4, *N* = 60) after one-way ANOVA statistical analysis when significance was set to *p* < 0.05.

**Cell type**	**Gene function**					
	**Senescence marker**	**Cell cycling**	**Growth factor**	**Anti-apoptotic**	**Tumor suppressor**	**Apoptotic**
**AT-MSC**		Down-regulated AQP1	Down-regulated HGF		Up-regulated TP53	Up-regulated SOST
**BM-MSC**	Up-regulated GLB1	Up-regulated AQP1 CSF2 CTGF Down-regulated GLI3	Up-regulated FGF1 FGF18 PDGFD TGFA VEGFA Down-regulated FGF5		Up-regulated CDKN1A GDF6 LOC100146270	Up-regulated CASP3 CASP8 SOST
**DF**		Up-regulated AQP1 COMP Down-regulated CCND1 PCNA	Up-regulated FGF18 IGFBP5 Down-regulated EPGN HGF PDGFD	Up-regulated CLU Down-regulated BCL2	Up-regulated GDF6 Down-regulated LOC100146270 TP53	Down-regulated CASP3

* *All listed genes were significantly affected by donor age within the given cell type*.

† *AT, adipose tissue; BM, bone marrow; MSC, mesenchymal stem cells; DF, dermal fibroblasts. Red marked genes indicate an up-regulation in gene expression with increasing donor age. Blue marked genes indicate a down-regulation in gene expression with increasing donor age*.

**Figure 5:**
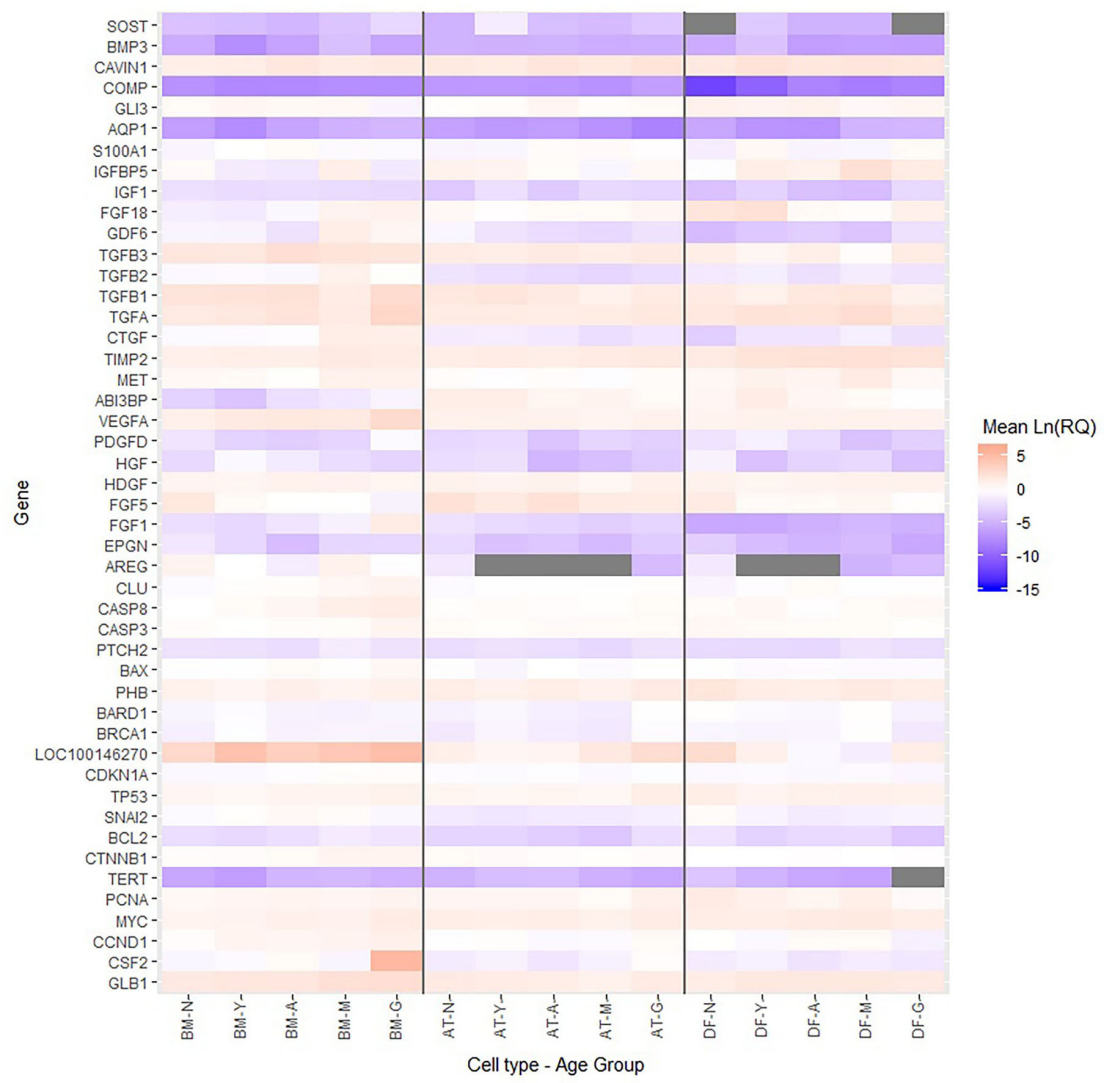
Relative levels of steady state mRNA for targeted gene loci. Heatmap showing the Ln(RQ) levels of gene expression by color change between average levels in bone marrow (BM)- and adipose tissue (AT)-derived mesenchymal stem cells (MSCs) and dermal fibroblasts (DF) from horses in five different age groups (*n* = 4 horses per age group per cell type) when grown in T75 cm^2^ culture flasks with normal expansion medium containing DMEM, 10% FBS, and 1% P/S and harvested at approximately 80% confluence. Gray boxes indicate no detection of gene expression. N, newborn; Y, yearling; A, adult; M, middle-aged; G, geriatric.

Multiple growth factors (FGF1, FGF18, GDF6, PDGFD, TGFA, and VEGFA) were up-regulated in BM-MSCs from old horses compared to young horses. On the other hand, fibroblast growth factor 5 (FGF5) was found at lower levels in BM-MSCs from geriatric horses compared to newborns (*p* = 0.02), and in higher levels in AT-MSCs compared to BM-MSCs (*p* = 0.008). Cell cycle regulators cyclin D (CCND1) and proliferating cell nuclear antigen (PCNA) were not significantly affected by donor age in either of the two stem cell types, but were down-regulated in DFs from geriatric horses (*p* = 0.01 and *p* < 0.0001, respectively). In general, cyclin D was found at higher levels in BM-MSCs compared to AT-MSCs and DFs (*p* < 0.003), whereas PCNA was found at higher levels in DFs compared to AT-MSCs (*p* < 0.0001) and BM-MSCs (*p* = 0.0004). For positive regulators of cell proliferation colony stimulating factor 2 (CSF2) was up-regulated in BM-MSCs from geriatric horses compared to all other age groups (*p* < 0.0005), whereas GLI family zinc finger 3 (GLI3) was down-regulated in geriatric BM-MSCs (*p* ≤ 0.02) (Figure 5, Table 3).

For BM-MSCs, expression of the senescence marker β-galactosidase (GLB1) was significantly higher in geriatric horses compared to newborns (*p* = 0.002), yearlings (*p* = 0.02), and adult horses (*p* = 0.03), and in middle-aged compared to newborn horses (*p* = 0.01). GLB1 was not significantly affected by donor age in AT-MSCs or DFs. Higher GLB1 expression was seen in BM-MSCs compared to AT-MSCs and DFs in geriatric horses (*p* = 0.001 and *p* = 0.0003, respectively) (Figure 5, Table 3). Steady state mRNA levels of aquaporin (AQP1), which encodes a membrane-associated water channel protein, were low relative to the positive control sample and changed as a function of age in all three cell types, increasing in older horses for BM-MSCs and DFs while decreasing in AT-MSCs.

**Figure 6:**
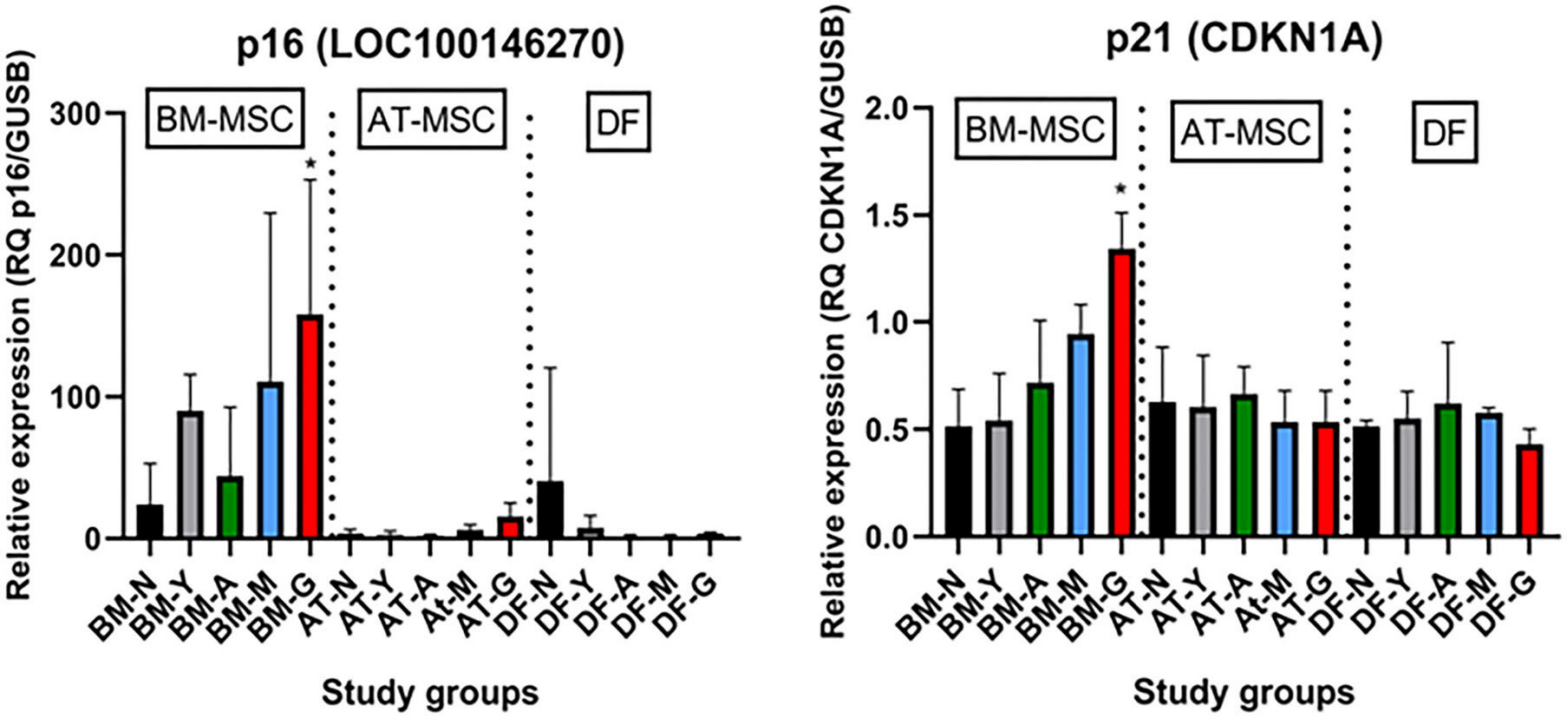
Age- and cell type-dependent p16 and p21 differences. Bar plots showing mean relative gene expression (RQ) of p16 and p21 steady state mRNA in monolayer cultures of bone marrow (BM)- and adipose tissue (AT)-derived mesenchymal stem cells (MSC) and dermal fibroblasts (DF) from horses in five different age groups (*n* = 4, *N* = 60) relative to the positive control. *Indicates significant difference between geriatric and newborn age group for BM-MSCs (*p* < 0.05). N, newborn (black); Y, yearling (gray); A, adult (green); M, middle-aged (blue); G: geriatric (red).

As shown in Figure 6, the tumor suppressors p16 (LOC100146270) and p21 (CDKN1A) were both up-regulated in geriatric BM-MSCs compared to newborn horses (*p* = 0.01 and *p* = 0.02, respectively). Moreover, p16 and p21 showed higher levels in BM-MSCs than in AT-MSCs (*p* ≤ 0.02) or DFs (*p* ≤ 0.0002). For AT-MSCs, the tumor suppressor p53 (TP53) had higher expression in geriatric horses compared to newborn (*p* = 0.002) or yearlings (*p* = 0.008). The pro-apoptotic genes caspase 3 (CASP3) and caspase 8 (CASP8) were up-regulated in BM-MSCs from older horses (*p* ≤ 0.003).

## Discussion

Results from the present study show that *in vitro* proliferation percentages of plastic-adherent equine BM- and AT-MSCs were stable through adult ages, but decreased in samples collected from geriatric donors. These findings support the hypothesis generally consistent with Alicka et al., who showed a shorter population doubling time in AT-MSCs from horses below 5 years of age compared to horses above 15 years of age (18). Additionally, Vidal et al. reported no difference in proliferative capacity of BM-MSCs from horses below 5 years of age (16). Schröck et al. reported a heterogeneous population of equine BM-MSCs with a decreasing population of cells with maximum proliferation speed with increasing donor age (20). Schröck's study, however, had a limited study population and was not designed to provide kinetics or thresholds with regards to donor age. The current study extends our understanding in horses by analyzing additional age groups, and interestingly shows that difference in equine BM- and AT-MSC cellular proliferation were only seen in pair-wise comparisons involving geriatric horses. No difference in cellular proliferation rates were observed in MSCs from horses below 18 years of age. This finding is broadly consistent with the gene expression data, where the majority of age-related changes in steady state mRNA levels were seen in comparisons involving geriatric horses. Decline in proliferative capacity with increasing donor age is similar to previous reports in other species (12–15). However, the pair-wise comparisons from horses stand in contrast with other species where differences in proliferation were more progressive and reported already in 8-year-old monkeys (12) and in humans above 40 years of age (32).

Cellular proliferation of human AT-MSCs have previously been reported to be less prominently affected by donor age compared to BM-MSCs (33). This was not observed in the present study, where equine BM- and AT-MSCs showed similar cellular proliferation in all age groups and were equally affected by donor age. Together, this may heighten the relevance of BM-MSCs for autologous treatments, as they have shown a higher therapeutic potential for cartilage and bone injuries (6, 34, 35). On the other hand, less age related gene expression changes were seen in AT-MSCs with the selected biomarker panel compared to BM-MSCs or DFs. It is likely that the cells have other age-related expression changes outside the selected biomarker panel as the phenotypic cellular proliferation was equal for AT- and BM-MSCs across age groups.

Our finding that DFs have generally higher cellular proliferation compared to BM-and AT-MSCs is supported by previous studies where DFs have been shown to be highly sensitive to mitogenic stimuli and readily expand in adherent monolayer cultures (19). The decrease in cellular proliferation with increasing donor age for all three cell types is broadly consistent with a general decline in the proliferative capacity of equine primary cells isolated from donor horses with increasing age.

Molecular mechanisms responsible for the observed decrease in equine cellular proliferation in samples collected from geriatric donors could potentially include a decrease in growth factors and their receptors, a decrease in pro-cycling molecules, and/or an increase in cell cycle arrest genes and apoptotic factors. Similar to previous studies, our data demonstrated that the tumor suppressor genes p53, p21 and p16 were up-regulated in MSCs from old donors (12, 14, 18). p16 and p21 inhibit DNA replication by inhibiting cyclin dependent kinases and are considered markers of senescence (36). p53 is a transcription factor for p21 and can induce transcription of apoptosis-associated genes like BAX, which leads to activation of CASP3, another apoptotic factor (37). Interestingly, increased p53 expression in old AT-MSCs did not result in detectable up-regulation of p21 or BAX in the geriatric AT-MSCs, which might be due to a lack of phosphorylation of p53 and a resulting rapid degradation. An increase in the apoptotic pro-factor CASP8 can also cause up-regulation of CASP3 (37), possibly explaining why both CASP8 and CASP3 were up-regulated in BM-MSCs from geriatric horses.

Taken together, the age related up-regulation of tumor suppressors and apoptotic factors could explain the lower cellular proliferation seen in aged horses. This supports a model of more cells being growth arrested rather than progressing through the S-phase of the cell cycle and incorporating EdU, which is consistent with previous studies where increased G1/G0-arrest and prolonged time in S-phase have been reported in MSCs from aged donors (12, 18).

GLB1 activity is an additional marker of senescence, with higher gene expression in senescent cells being reported (38). In BM-MSCs, an age related increase in GLB1 expression was seen in geriatric horses corresponding well with previous studies showing accelerated cellular senescence with increasing donor age (15, 18).

Interestingly, multiple growth factors actually displayed higher steady state mRNA levels in BM-MSCs from geriatric horses. Age-dependent growth factor results are variable in previous rodent studies (13, 39, 40), but the current data suggests that the decreased proliferation observed in BM-MSCs from geriatric horses may be more due to an up-regulation in tumor-suppressors and apoptotic factors than a decrease in growth factors or pro-cycling factors. It is moreover possible that aged BM-MSCs are less responsive to secreted growth factors, consistent with studies investigating relationships between growth factor activity and inflammation in aging (41).

Potential age-dependent differences in cellular differentiation potential, as well as production of paracrine factors to influence the patient's immune response or endogenous progenitor cells relevant to cell-therapy application in horses were not investigated in the current experiments, but will be important parameters to assess in future studies. Another parameter not addressed was potential gender differences in the proliferation of equine MSCs as a function of age.

Primary cells were collected from female and male horses in each age group, but not with even distribution (Table 1). A relevant study in mice comparing *in vitro* MSC proliferation as a function of gender did not observe any differences (42). All donor horses in the study were in apparent good health for their age with a few exceptions in older animals (Table 1). Certain systemic health parameters could affect cell biology parameters including proliferation. The impact of specific systemic illnesses on MSCs, as in the case of the 25-year-old lymphoma horse in the current sample set, will require additional research. Primary cell lines in the different age groups did consist of both paired and non-paired samples. Ideally, the study would have been conducted solely with paired samples as inter-animal variation is an important consideration (43). To control for this factor, the data were treated statistically as non-paired and horse number was added to the statistical models.

Evidence for trilineage differentiation potential is apparent in equine BM- and AT-MSCs isolated using the techniques applied in this study (data not shown). Unfortunately, uniform parameters to validate “stemness” of equine MSCs with cell surface molecular markers has not been established (44) and relevant antibodies for equine cell isolates remain limited (45–47).

In conclusion, all cells independent of age group were able to be expanded to passage 4, but the proliferation of plastic adherent equine BM- and AT-MSCs declined significantly in the geriatric age group. High *in vitro* cellular proliferation was seen in BM- and AT-MSCs from horses below 18 years of age. The cellular proliferation of BM- and AT-MSCs was similar in all age groups and they were equally affected by donor age. Underlying gene expression changes related to proliferation and aging showed a primary up-regulation in tumor suppressors, apoptotic genes, and multiple growth factors in MSCs from old horses, and a down-regulation of some pro-cycling genes depending on cell type. MSC differentiation potential, as well as the capacity to mediate inflammatory processes and other paracrine functions will be important parameters to consider with further research on donor age as a variable in cell-based therapies for horses.

## Data Availability Statement

The original contributions presented in the study are included in the article/Supplementary Materials, further inquiries can be directed to the corresponding author/s.

## Ethics Statement

The animal study was reviewed and approved by the ethical guidelines of animal research at the University of Copenhagen and the University of Kentucky. Written informed consent was obtained from the owners for the participation of their animals in this study.

## Author Contributions

JB, JM, and LB were responsible for the study design, harvest of cells, and for obtaining funding. Acquisition of data, data analyses and interpretation, and writing the first draft of the manuscript was done by JB. All authors were involved in critical revision of the manuscript and final approval.

## Conflict of Interest

The authors declare that the research was conducted in the absence of any commercial or financial relationships that could be construed as a potential conflict of interest.

## Supplementary Material

Supplementary Table 1 Table with Ct and Ln(RQ) data used for statistical analysis and the heatmap.

## References

[1] Frisbie DD Smith RKW Clinical update on the use of mesenchymal stem cells in equine orthopaedics. Equine Vet J. (2010) 42:86–9. 10.2746/042516409X477263 20121921

[2] Schnabel LV Lynch ME van der Meulen MCH Yeager AE Kornatowski MA Nixon AJ. Mesenchymal stem cells and insulin-like growth factor-I gene-enhanced mesenchymal stem cells improve structural aspects of healing in equine flexor digitorum superficialis tendons. J Orthop Res. (2009) 27:1392–8. 10.1002/jor.20887 19350658

[3] Pfeiffenberger M Bartsch J Hoff P Ponomarev I Buttgereit F Gaber T. Hypoxia and mesenchymal stromal cells as key drivers of initial fracture healing in an equine *in vitro* fracture hematoma model. PLoS ONE. (2019) 14:e0214276. 10.1371/journal.pone.0214276 30947253 PMC6449067

[4] Dexheimer V Frank S Richter W. Proliferation as a requirement for *in vitro* chondrogenesis of human mesenchymal stem cells. Stem Cells Dev. (2012) 21:2160–9. 10.1089/scd.2011.0670 22229819 PMC3411365

[5] Kang BJ Ryu HH Park SS Koyama Y Kikuchi M Woo HM. Comparing the osteogenic potential of canine mesenchymal stem cells derived from adipose tissues, bone marrow, umbilical cord blood, and Wharton's jelly for treating bone defects. J Vet Sci. (2012) 13:299–310. 10.4142/jvs.2012.13.3.299 23000587 PMC3467406

[6] Adam EN Janes J Lowney R Lambert J Thampi P Stromberg A. Chondrogenic differentiation potential of adult and fetal equine cell types. Vet Surg. (2019) 48:375–87. 10.1111/vsu.13183 30801754

[7] Korchunjit W Laikul A Taylor J Watchrarat K Ritruechai P Supokawej A Characterization and allogeneic transplantation of equine bone marrow-derived multipotent mesenchymal stromal cells collected from cadavers. J Equine Vet Sci. (2019) 73:15–23. 10.1016/j.jevs.2018.11.004

[8] Shojaee A Parham A. Strategies of tenogenic differentiation of equine stem cells for tendon repair: current status and challenges. Stem Cell Res Ther. (2019) 10:1–13. 10.1186/s13287-019-1291-0 31215490 PMC6582602

[9] Zahedi M Parham A Dehghani H Mehrjerdi HK. Equine bone marrow-derived mesenchymal stem cells: optimization of cell density in primary culture. Stem Cell Investig. (2018) 5:1–8. 10.21037/sci.2018.09.01 30498742 PMC6232052

[10] Sareen N Sequiera GL Chaudhary R Abu-El-Rub E Chowdhury SR Sharma V Early passaging of mesenchymal stem cells does not instigate significant modifications in their immunological behavior. Stem Cell Res Ther. (2018) 9:1–11. 10.1186/s13287-018-0867-4 29720263 PMC5930635

[11] Hatsushika D Muneta T Nakamura T Horie M Koga H Nakagawa Y. Repetitive allogeneic intraarticular injections of synovial mesenchymal stem cells promote meniscus regeneration in a porcine massive meniscus defect model. Osteoarthr Cartil. (2014) 22:941–50. 10.1016/j.joca.2014.04.028 24795274

[12] Yu JM Wu X Gimble JM Guan X Freitas MA Bunnell BA. Age-related changes in mesenchymal stem cells derived from rhesus macaque bone marrow. Aging Cell. (2011) 10:66–79. 10.1111/j.1474-9726.2010.00646.x 20969724 PMC4339051

[13] Asumda FZ Chase PB. Age-related changes in rat bone-marrow mesenchymal stem cell plasticity. BMC Cell Biol. (2011) 12:44. 10.1186/1471-2121-12-44 21992089 PMC3204286

[14] Zhou S Greenberger JS Epperly MW Goff JP Adler C Leboff MS. Age-related intrinsic changes in human bone-marrow-derived mesenchymal stem cells and their differentiation to osteoblasts. Aging Cell. (2008) 335–43. 10.1111/j.1474-9726.2008.00377.x 18248663 PMC2398731

[15] Stenderup K Justesen J Clausen C Kassem M. Aging is associated with decreased maximal life span and accelerated senescence of bone marrow stromal cells. Bone. (2003) 33:919–26. 10.1016/j.bone.2003.07.005 14678851

[16] Vidal MA Kilroy GE Johnson JR Lopez MJ Moore RM Gimble JM. Cell growth characteristics and differentiation frequency of adherent equine bone marrow-derived mesenchymal stromal cells: Adipogenic and osteogenic capacity. Vet Surg. (2006) 35:601–10. 10.1111/j.1532-950X.2006.00197.x 17026544

[17] Vidal MA Kilroy GE Lopez MJ Johnson JR Moore RM Gimble JM. Characterization of equine adipose tissue-derived stromal cells: adipogenic and osteogenic capacity and comparison with bone marrow-derived mesenchymal stromal cells. Vet Surg. (2007) 36:613–22. 10.1111/j.1532-950X.2007.00313.x 17894587

[18] Alicka M Kornicka-Garbowska K Kucharczyk K Kepska M R?cken M Marycz K. Age-dependent impairment of adipose-derived stem cells isolated from horses. Stem Cell Res Ther. (2020) 11:4. 10.1186/s13287-019-1512-6 31900232 PMC6942290

[19] Thampi P Dubey R Lowney R Adam EN Janse S Wood CL. Effect of skeletal paracrine signals on the proliferation of interzone cells. Cartilage. (2019) 25:1–13. 10.1177/1947603519841680 31023058 PMC8804777

[20] Schröck C Eydt C Geburek F Kaiser L Felicitas P Burk J. Bone marrow-derived multipotent mesenchymal stromal cells from horses after euthanasia. Vet Med Sci. (2017) 3:239–51. 10.1002/vms3.74 29152317 PMC5677777

[21] ter Huurne M Schelbergen R Blattes R Blom A De Munter W Grevers LC. Antiinflammatory and chondroprotective effects of intraarticular injection of adipose-derived stem cells in experimental osteoarthritis. Arthritis Rheum. (2012) 64:3604–13. 10.1002/art.34626 22961401

[22] Pezzanite LM Fortier LA Antczak DF Cassano JM Brosnahan MM Miller D. Equine allogeneic bone marrow-derived mesenchymal stromal cells elicit antibody responses *in vivo*. Stem Cell Res Ther. (2015) 6:1–11. 10.1186/s13287-015-0053-x 25889095 PMC4414005

[23] Joswig A Mitchell A Cummings KJ Levine GJ Gregory CA Smith R III. Repeated intra-articular injection of allogeneic mesenchymal stem cells causes an adverse response compared to autologous cells in the equine model. Stem Cell Res Ther. (2017) 8:1–11. 10.1186/s13287-017-0503-8 28241885 PMC5329965

[24] Whitworth DJ Banks TA Stem cell therapies for treating osteoarthritis: prescient or premature? Vet J. (2014) 202:416–24. 10.1016/j.tvjl.2014.09.024 25457267

[25] Bundgaard L Stensballe A Elbæk KJ Berg LC. Mapping of equine mesenchymal stromal cell surface proteomes for identification of specific markers using proteomics and gene expression analysis: an *in vitro* cross-sectional study. Stem Cell Res Ther. (2018) 9:1–10. 10.1186/s13287-018-1041-8 30359315 PMC6202851

[26] Mienaltowski MJ Huang L Stromberg AJ Macleod JN. Differential gene expression associated with postnatal equine articular cartilage maturation. BMC Musculoskelet Disord. (2008) 9:1–14. 10.1186/1471-2474-9-149 18986532 PMC2585085

[27] Hestand MS Kalbfleisch TS Coleman SJ Zeng Z Liu J Orlando L. Annotation of the protein coding regions of the equine genome. PLoS ONE. (2015) 10:e0124375. 10.1371/journal.pone.0124375 26107351 PMC4481266

[28] Ramakers C Ruijter JM Lekanne RH Moorman AFM. Assumption-free analysis of quantitative real-time polymerase chain reaction (PCR) data. Neorosci Lett. (2003) 339:62–6. 10.1016/S0304-3940(02)01423-4 12618301

[29] Andersen CL Jensen JL Ørntoft TF. Normalization of real-time quantitative reverse transcription-PCR data: a model-based variance estimation approach to identify genes suited for normalization, applied to bladder and colon cancer data sets. Cancer Res. (2004) 64:5245–50. 10.1158/0008-5472.CAN-04-0496 15289330

[30] Olwagen CP Adrian P V Madhi SA. Performance of the Biomark HD real-time qPCR System (Fluidigm) for the detection of nasopharyngeal bacterial pathogens and *Streptococcus pneumoniae* typing. Sci Rep. (2019) 9:6494. 10.1038/s41598-019-42846-y 31019272 PMC6482308

[31] Livak JK Schmittgen T. Analysis of relative gene expression data using real-time quantitative PCR and the 2–ΔΔCT method. Methods. (2001) 25:404–8. 10.1006/meth.2001.1262 11846609

[32] Stolzing A Jones E Mcgonagle D Scutt A. Age-related changes in human bone marrow-derived mesenchymal stem cells: consequences for cell therapies. Mech Ageing Dev. (2008) 129:163–73. 10.1016/j.mad.2007.12.002 18241911

[33] Chen H Lee M Chen C Chuang S. Proliferation and differentiation potential of human adipose-derived mesenchymal stem cells isolated from elderly patients with osteoporotic fractures. J Cell Mol Med. (2012) 16:582–92. 10.1111/j.1582-4934.2011.01335.x 21545685 PMC3822933

[34] Vidal MA Robinson SO Lopez MJ Paulsen DB Borkhsenious O Johnson JR. Comparison of chondrogenic potential in equine mesenchymal stromal cells derived from adipose tissue and bone marrow. Vet Surg. (2008) 37:713–24. 10.1111/j.1532-950X.2008.00462.x 19121166 PMC2746327

[35] Park SH Sim WY Min BH Yang SS Khademhosseini A Kaplan DL. Chip-based comparison of the osteogenesis of human bone marrow- and adipose tissue-derived mesenchymal stem cells under mechanical stimulation. PLoS ONE. (2012) 7:1–11. 10.1371/journal.pone.0046689 23029565 PMC3460891

[36] Beauséjour CM Krtolica A Beause CM Galimi F Narita M Lowe SW. Reversal of human cellular senescence: roles of the p53 and p16 pathways. EMBO J. (2003) 22:4212–22. 10.1093/emboj/cdg417 12912919 PMC175806

[37] Jesenberger V Jentsch S. Deadly encounter: ubiquitin meets apoptosis. Nat Rev Mollecular Cell Biol. (2002) 3:112–21. 10.1038/nrm731 11836513

[38] Lee BY Han JA Im JS Morrone A Johung K Goodwin C. Senescence-associated β-galactosidase is lysosomal β-galactosidase. Aging Cell. (2006) 5:187–95. 10.1111/j.1474-9726.2006.00199.x 16626397

[39] Wilson A Shehadeh LA Yu H Webster KA. Age-related molecular genetic changes of murine bone marrow mesenchymal stem cells. BMC Genomics. (2010) 11:229. 10.1186/1471-2164-11-229 20374652 PMC2873471

[40] Zheng H Martin JA Duwayri Y Falcon G Buckwalter JA. Impact of aging on rat bone marrow-derived stem cell chondrogenesis. J Geriontol A Biol Sci Med Sci. (2007) 62:136–48. 10.1093/gerona/62.2.136 17339639

[41] Masternak MM Bartke A. Growth hormone, inflammation and aging. Pathobiol Aging Age Relat Dis. (2012) 2:17293. 10.3402/pba.v2i0.17293 22953033 PMC3417471

[42] Katsara O Mahaira LG Iliopoulou EG Moustaki A Antsaklis A Loutradis D. Effects of donor age, gender, and *in vitro* cellular aging on the phenotypic, functional, and molecular characteristics of mouse bone marrow-derived mesenchymal stem cells. Stem Cells Dev. (2011) 20:1549–61. 10.1089/scd.2010.0280 21204633

[43] Carter-Arnold JL Neilsen NL Amelse LL Odoi A Dhar MS. *In vitro* analysis of equine, bone marrow-derived mesenchymal stem cells demonstrates differences within age- and gender-matched horses. Equine Vet J. (2014) 46:589–95. 10.1111/evj.12142 23855680

[44] De Schauwer C Meyer E Van de Walle GR Van Soom A. Markers of stemness in equine mesenchymal stem cells: a plea for uniformity. Theriogenology. (2011) 75:1431–43. 10.1016/j.theriogenology.2010.11.008 21196039

[45] Radcliffe CH Flaminio MJBF Fortier LA. Temporal analysis of equine bone marrow aspirate during establishment of putative mesenchymal progenitor cell populations. Stem Cells Dev. (2010) 19:269–81. 10.1089/scd.2009.0091 19604071 PMC3138180

[46] De Schauwer C Piepers S Van de Walle GR Demeyere K Hoogewijs MK Govaere JLJ. In search for cross-reactivity to immunophenotype equine mesenchymal stromal cells by multicolor flow cytometry. Cytom Part A. (2012) 81A:312–23. 10.1002/cyto.a.22026 22411893

[47] Ibrahim S Saunders K Kydd JH Lunn DP Stainbach F. Screening of anti-human leukocyte monoclonal antibodies for reactivity with equine leukocytes. Vet Immunol Immunopathol. (2007) 119:63–80. 10.1016/j.vetimm.2007.06.034 17707518

